# The Programme Is on Trial

**Published:** 1898-08

**Authors:** 


### THE PROGRAMME IS ON TRIAL.

THE programme of the U. S. V. M. A. will be on trial at Omaha. After much consideration this year it has been planned almost wholly in the interest of the everyday practitioner, and the attendance and interest of this very large proportion of the active veterinarians all over the land will largely determine how far this plan will be followed out in future meetings of the association. Those who have many times contended that the meetings were almost wholly taken up with subjects of a general character, veterinary sanitary problems, infectious and contagious diseases, to the exclusion of the more practical ones of everyday import to the average veterinarian are certainly sure of abundant entertainment and profitable subjects for consideration at Omaha. Let this long-neglected class pour out in unusual numbers at Omaha, and future programmes will be solved. If not, then we must look only for local interest in national meetings and the development of kindred associations for the consideration of the broader and truly national problems of veterinary medicine.

